# Retention of allied health professionals in rural New South Wales: a thematic analysis of focus group discussions

**DOI:** 10.1186/1472-6963-12-175

**Published:** 2012-06-22

**Authors:** Sheila Keane, Michelle Lincoln, Tony Smith

**Affiliations:** 1 University Centre for Rural Health, University of Sydney, PO Box 3074, Lismore, NSW, Australia; 2 Faculty of Health Sciences, The University of Sydney, Lidcombe, NSW, Australia; 3 University Department of Rural Health – Northern NSW, University of Newcastle, Tamworth, NSW, Australia

## Abstract

**Background:**

Uneven distribution of the medical workforce is globally recognised, with widespread rural health workforce shortages. There has been substantial research on factors affecting recruitment and retention of rural doctors, but little has been done to establish the motives and conditions that encourage allied health professionals to practice rurally. This study aims to identify aspects of recruitment and retention of rural allied health professionals using qualitative methodology.

**Methods:**

Six focus groups were conducted across rural NSW and analysed thematically using a grounded theory approach. The thirty allied health professionals participating in the focus groups were purposively sampled to represent a range of geographic locations, allied health professions, gender, age, and public or private work sectors.

**Results:**

Five major themes emerged: personal factors; workload and type of work; continuing professional development (CPD); the impact of management; and career progression. ‘Pull factors’ favouring rural practice included: attraction to rural lifestyle; married or having family in the area; low cost of living; rural origin; personal engagement in the community; advanced work roles; a broad variety of challenging clinical work; and making a difference. ‘Push factors’ discouraging rural practice included: lack of employment opportunities for spouses; perceived inadequate quality of secondary schools; age related issues (retirement, desire for younger peer social interaction, and intention to travel); limited opportunity for career advancement; unmanageable workloads; and inadequate access to CPD. Having competent clinical managers mitigated the general frustration with health service management related to inappropriate service models and insufficient or inequitably distributed resources. Failure to fill vacant positions was of particular concern and frustration with the lack of CPD access was strongly represented by informants.

**Conclusions:**

While personal factors affecting recruitment and retention of allied health study participants were similar to doctors, differences also existed. Allied health professionals were attracted by advanced work roles in a context of generalist practice. Access to CPD and inequitable resource distribution were strong ‘push’ factors in this group. Health policy based on the assumption of transferability between professions may be misguided.

## Background

It is well established that there is a global shortage of health professionals working in rural areas. The World Health Organisation reported that, while 50% of the population worldwide lives rurally, only 38% of nurses and 24% of the medical workforce practice rurally [1]. Based on 2006 Australian census data, 31% of Australians live rurally while 19% of physicians and 31% of nurses work in rural locations. In the 2006 census, just 23% of the ten allied health (AH) professions counted in the census were working in a rural setting. Recent research on intention to leave [2,3] and an aging demographic [4] suggest a substantial and imminent exodus of AH professionals from rural practice in Australia [5].

Considerable international research has shown that recruitment and retention of rural doctors is influenced by personal factors such as rural background, career intent and service orientation [6]. Rural training, rural curricular content, and rural student clinical rotations also influence retention of rural doctors [7]. While the role of continuing professional education is less clear, [8] financial incentives, career opportunities and access to personal and professional support have also featured as factors influencing retention of rural doctors [9]. A number of intervention strategies based on this body of evidence are being undertaken internationally to improve medical workforce distribution [10].

Retention of rural nurses is less well studied but there appear to be some differences in motives to remain in rural practice compared with doctors. For example, relational aspects of work (e.g. working in teams, peer support and recognition) have been cited as reasons nurses choose to remain in rural practice [11]. Management approaches that provide guidance, emotional support and appreciation also play an important role in job satisfaction for rural nurses [12]. Lack of management support has been associated with intention to leave [13].

Allied health professionals play important roles in diagnosis, treatment, prevention and rehabilitation of illness or injury [14]. Retaining a rural AH workforce is essential to ensure access to AH services for rural residents and minimize the high costs of staff turnover [15]. Yet, very little research has examined rural workforce retention specifically in the AH professions [16,17].

This study builds on the results of the Rural Allied Health Workforce (RAHW) survey conducted in 2008, which targeted allied health professionals in 21 occupational categories who were practicing in regional, rural and remote areas of NSW (ASGC-RA 2 to 5) [18]. The RAHW questionnaire has been described in detail in a previously published article [19] and included questions on personal background, work practices and education, as well as questions exploring a range of factors relating to recruitment and retention.

Among the findings is that gender differences may impact on choice of practice location, and respondents were attracted by personal factors as well as type of work. Forty two percent intended leaving their job in the next 5 years [20]. As with all questionnaire based research, participant answers were restricted to agreement with statements and categorical responses. The RAHW study provided statistical information about the rural allied health workforce and potential issues affecting retention, however in depth exploration and explanation of these issues was not possible using this research method. Hence, this qualitative study aimed to add depth to RAHW survey findings using focus group methodology, and to further identify factors affecting recruitment and retention of the rural AH workforce.

## Methods

After obtaining ethics approval from the University of Sydney, six semi-structured focus groups were conducted in regional centres across rural New South Wales over a 12 month period. Participants were drawn from a pool of volunteers who had answered the 2008 RAHW survey, and subjects were purposively selected to represent a range of geographic locations, allied health professions, gender, age, and public or private work sectors. One of the later focus groups targeted younger participants using snowball sampling. An experienced AH professional who was previously unknown to participants conducted the focus groups, clarifying points and ensuring that each participant contributed.

Focus groups were used to develop a consensus on relevant opinions and attitudes on an array of foci, and interview questions were broad to allow for unanticipated themes to emerge. In each 90 minute focus group, participants were asked: (a) What brought you to this area? (b) What brought you to your current job? (c) What might make you or your colleague want to leave your job? and (d) What might make you want to leave the area? Opportunity for further comment was provided in the final 20 minutes of each session.

A final focus group was used to verify that saturation had been achieved and that data-generated themes had been clearly described. Because of early saturation on the impact of personal factors on attraction and retention, questions in this focus group were more focussed on professional issues such as: (a) Are there things about your job that make you want to stay working there? (b) How important is CPD for you? (c) Would you leave your job to pursue a better career path?

Verbatim transcripts of interviews were checked against the audio recording for accuracy. A grounded theory approach was used, concurrently collecting and analyzing data, [21] and emerging themes were tested and clarified in subsequent focus groups. Transcripts were analysed thematically [22] by creating coding nodes for common themes and subthemes using NVivo 8 [23]. Two of the 6 transcripts were independently coded by the co-authors to validate the nodes [24]. Nodes were modified for consistency between transcripts and added as new themes emerged [25]. No new nodes appeared after 6 focus groups.

A printed summary of emergent themes was presented to participants for validation. They did not correct or identify any unaccounted for themes.

In this paper, all quotations from participants are indicated in italics, with the respondent identified by group number, profession, gender and age. Abbreviations of each profession are listed in Table 1.

**Table 1:** Focus group participants

** Allied health discipline**	**Number participating**
Dietician (DT)	1
Optometrist (OPT)	1
Occupational therapist (OT)	5
Physiotherapist (PT)	7
Psychologist (PSY)	4
Radiographer (RAD)	2
Social worker (SW)	3
Counsellor, Social welfare worker * (SW)	2
Speech pathologist (SP)	2
Manager (MGR)	3

*The counsellor and social welfare worker were both acting in social work positions that could not be successfully recruited.

## Results

A total of 30 individuals participated in the six focus group interviews. The median age was 44 years (range 24 to 63 years), with a median of 15 years of experience (range 1 to 43 years). Twenty four (80%) were female, 5 (17%) were private practitioners and 9 (30%) often or always worked in sole practice settings. Professions represented in the sample are listed in Table 1.

Five major themes emerged: personal factors; workload and type of work; continuing professional development (CPD); the impact of management; and career progression. Some aspects of these themes favoured retention (‘pull factors’) and others increased intention to leave (‘push factors’) [26]. A summary of themes and subthemes confirmed by participants is presented in Table 2. 

**Table 2:** Focus group themes: Factors affecting recruitment and retention

**Theme**	**“Push” factor (leave job)**	**“Pull” factor (recruit/retain in job)**
Personal factors:	Lack of job opportunities for spouses	
	Perceived inadequate quality of secondary schools	Attraction to rural lifestyle
	Care for elderly parents	Good place to raise children
	Retirement	Married to a local resident
	Family members living in a metropolitan area	Rural origin/family in area
	For younger AHP’s:	Low cost of living
	Limited social opportunities	Personal engagement in the community
	Desire for adventure/travel	
Career progression:	Better career opportunities in metropolitan settings	NSW Health Award structure (accelerated promotion for new graduates in rural practice settings)
	Lower income, smaller market for rural private practitioners	Recognition by peers and others
	Rural senior positions unavailable or not open for recruitment	Advanced work roles
		Appropriate remuneration
Workload and type of work:	Unmanageable workload	Altruism, making a difference
	Crisis mode of service, reactive not preventive	Direct individual patient care
	Paperwork, reporting requirements	Generalist practice with advanced work roles - ‘specialist generalist’
		Challenge, variety and intellectual stimulation embedded in the job
Continuing Professional Development (CPD):	Limited access to CPD due to:	University campus in regional centres increases CPD access
	Lack of management support to attend CPD events	Access to CPD:
	Cost of travel	ameliorates professional isolation
	Expensive registrations (metropolitan courses)	is strongly linked with job satisfaction
	Time away from work	is essential for new graduates and isolated practitioners
	High workload demands	Assures that senior clinicians skills remain up to date
		Provides intellectual challenge and opportunities for career progression
The impact of management:	Perceived inequitable or inappropriate resource allocation	Supportive line managers
	Nurse managers	Support for CPD access
	Failure to recruit vacant positions	Clinical mentorship for new graduates
	Constant change	Flexible work hours
	Managers who are unresponsiveness to suggestions	Autonomy
	Feeling de-valued	Equitable resource allocation sufficient to deliver clinical services
	Ethical compromise – fiscal vs. clinical imperatives	Realistic estimate of workload capacity
	Move to private practice to escape public sector “management	

### Personal influences

Community engagement and personal relationships were powerful motivators for retention. Many participants either grew up rurally or were attracted to a rural lifestyle, and participants who were parents also felt that it was a good place to raise their children (Table 3). ‘Push’ factors in the personal domain included insufficient community infrastructure such as transportation, secondary schools, access to shops and jobs for spouses. Access to adequate accommodation was also a concern in more remote regions.

**Table 3:** Major theme and subthemes: Personal influences

** Subthemes**	**Examples**
Influence of family and children	*We were looking for the quiet, manageable, get the kids to all the things we wanted them to have the opportunity to do, and that's why we moved rurally. (G6-SP-Female-age 50)* *I was just after a change, a lifestyle change. A change of pace, as well. And I guess – well, all my family is in the city, so that’s been the hardest thing, being so far away. It might be a factor in making a consider moving, but we’ll see how that goes (G3-OT-Female-age 27)*
Rural lifestyle and rural origin	*I grew up in the country, I didn’t grow up in this area, though, so I always wanted to get back to the bush to work (G4-PSY-Female-age 36)*
Community support	*3 or 4 years ago I did a locum out here and thought that this community was a wonderful community and my colleagues were wonderful, and there was a kind of appreciation for what I thought was just doing my job that was different to metropolitan centres (G5-MGR-Female-age 44)*
Embeddedness in community life	*You know, 5 years ago I would have worked for 12 months and then moved on to find somewhere new, but I’m all settled now, and I’m happy where I am. (G2-OT-Female-age 33)*
Community infrastructure	*Schools* *We’re really happy with all the primary schools here… but the high schools are a bit of a worry. So we’ll really have to think about what high schools for the kids (G3-PSY-Male-age 46)* *Jobs* *I think it's one of the problems for people that have partners. This town has had a real hollowing out of what we might call white-collar positions over the last 15 years… Most public service offices have been moved out of the town and I think that makes it hard for couples to think about coming here. (G1- PSY-Female-age 57)* *Accommodation* *If someone's really interested in coming here they ask about the accommodation, and they ask about what does it have to offer, you know, sport or the arts or whatever, you know, their interests, what sort of groups are there and that sort of thing, because it is a lifestyle thing. (G5-MGR-Female-age 56)*

Several older participants said that they left their rural homes when young in order to practice in a metropolitan setting, returning for personal reasons after decades of being away. Younger AH professionals commonly left the region early in their career for travel or adventure, and also to find a better peer social environment.

I guess socially, too, there’s a bit more happening [in the city]. A lot of the younger staff at the hospital here, they all rotate through so you get to know them and then they leave, whereas – and everyone in X-ray – well, not everyone, but most of them are all quite a bit older and have kids and things… (G3-RAD-Female-age 23)

### Workload and type of work

While the desire to specialise was a ‘push’ factor for many early career AH professionals, older participants reported deriving job satisfaction from having a broad variety of clinical work (Table 4). 

**Table 4:** Major theme and subthemes: Workload and type of work

**Subthemes**	**Example**
Workload	*I'm doing two jobs and have been doing for two and a half months. Recruitment is happening and it's going, and I hit the wall and my manager said, “Keep on going,” and I said, “Can you just acknowledge how much extra – all you need to do is acknowledge it.” I'm still doing it and I'll still keep doing it, but if I wasn't going on maternity leave I'd be taking[stress] leave at the end of it because you can't do it. (G6-OT-Female-age28)*
Broad variety of clinical work	*With most professions… there’s a pressure on you to sort of specialize, and I’ve chosen to be a generalist so I like to be able to do everything from three-day-old babies with talipes to 105-year-old little old ladies… A broad range of practices. That’s how I ended up doing what I’m doing. (G4-PT-Male-age 57)*
Altruism and making a difference	*I discharged a patient yesterday, 19 years old, and he had cerebral palsy. When he came to me as a baby they were told that he would never walk or talk. Now he’s leaving school, walking independently, talks, communicates, he's got a traineeship. And I said to him, “I'm going to discharge you before I retire because you don't need physio anymore,” and they both started crying, him and his mom, and I gave them both a kiss and off they went. And later on in the day I got some lovely flowers. But that's what makes country practice. You won't get that in a big city. (G5-PT-Female-age57)*
Direct clinical work (managers)	*The clinical side of things, I think, is very rewarding. I'm actually administrative management, and I'm supposed to be solely that, and I cannot do it. I just cannot do it. Whenever I get an opportunity to do some clinical work I say “I'll do it. I'm free.” Because I'm sick of the desk thing. And it's because of the satisfaction with the clinical load. You need to have, I think, that satisfaction. The desk thing –I just get frustrated, I guess because the system is very hard to work within with the lack of resources, lack of funding, and I need the outlet to help me survive. I think treating the patients is why we're here. (G2-MGR-Female-age 44)*
Type of work and career progression	*I do think money comes into a bit, because you can't – in the city – it's not the only reason why you would go for another job at all, but I think it does come into it, because in the city, you know, you can specialize in seeing one type of child, or one type of disorder, and get paid much more than a rural clinician who has to be good at seeing it all. And that does become frustrating (G2-DT-Female- age 33)*

Managers were particularly keen to retain some contact with direct clinical practice. Large workloads, as well as bureaucracy, lack of management support, and inadequate access to continuing professional development (CPD) were frequently mentioned in relation to job dissatisfaction. Such was the level of dissatisfaction that some participants intended to leave their career, not just their job.

It's more about, you know, do you get the support in your job, do you get the support for professional development, can you manage the ridiculous case loads, all those things that put pressure on the job. I honestly think that if I left speech pathology in government health, I might do a little bit of private, but even then I think really for me to leave speech pathology in health, I think I would really have to be jack of it, and I'd do something else like work at a book store, because you know, I love health and I love my job, and I love lots of things about it. There's lots that I don't like. Lots of bureaucracy that I hate, but overall, I like my profession and that's why I'm here, and if I left it, I think I'd be leaving for good. (G2-SP-Female-age 31)

Clinicians also found it very stressful to have to prioritise patients to manage heavy workloads, resulting in long waiting lists.

Because it's you that gets that phone call from the mom who says, “My child can't – or in my case, doesn't - eat anything.” and it's not the management that gets that phone call. Like you have to say, “Sorry, you'll have to wait a year until I see you”… it is the clinician that is the coal face, seeing that person, and you see them when you're down at the shopping mall and you see that kid hobbling along and you haven't dealt with it, yeah. I think that's – and it just grinds you down. (G2-DT-Female-age 32)

### The importance of access to CPD

The issue of limited CPD access was raised (unprompted) within the first 5 min of all six focus groups. The topic often elicited a forceful expression of frustration and job dissatisfaction. CPD needs varied by stage of career. Older participants sought CPD access for professional stimulation, as a mechanism to remain up to date with evidence based clinical practice and as a remedy for professional isolation. In contrast, most young participants sought to consolidate newly acquired clinical skills and some followed a career intention toward specialization.

All participants saw CPD access and professional mentorship as being essential for new graduates. There was a mixed response to obtaining CPD delivered online, partly due to variation in the availability of equipment and technical support. Younger participants were more comfortable with online learning.

Access to CPD was limited by cost and travel time, as well as unmanageable workloads that were increased by taking time off to attend a course (Table 5). Since CPD access also served as a remedy for professional isolation, face to face courses were preferred by most participants. 

**Table 5:** Major theme and subthemes: Access to continuing professional development (CPD)

**Subthemes**	**Example**
Cost & travel time	*You have to pay for it yourself and it makes it really difficult to do professional development You end up begrudging the fact that you want to do it, because you're going to have to take three or four days annual leave to do it, and plus be $400 out of pocket for doing it. You maintain your skills and you stay in that job. So those type of things have made a huge difference to job satisfaction (G2-Female-SP-age 32)*
Workload & Management support	*They're not going to stop people having operations or being admitted to hospital because there are no allied health services. And then they turn around and say, “Because you're short-staffed, we can't allow your staff to go off and do CPD.” So it then becomes a perpetual cycle that – You know, you do lose staff over it. (G6-MGR-Female-age 51)*
Support for isolated practitioners	*I think we kind of sell it short by just calling it professional development, because it's not always just that. It's networking, it's support, it's all these different things that happen during that day, and I've heard people say, “The most useful thing about that day was the lunch break because I got to sit down and talk to other people.” (G6-SW-Male-age 30)*
Regional Networks	*I've worked as a sole therapist in several places, and to be honest I don't feel as much a sole therapist as I have anywhere else because we do a lot of networking and our CPD and things, and it just makes a huge difference to know that you can pick up the phone and talk to someone if you need to. (G2-OT-Female-age 33)*
On-line education	*Well, we know that learning is best achieved in interactive and experiential ways, and this switch through to online learning, just flies in the face of it. (G1-PSY-Female-age 57)* *And it's great, we go to the facilities out at this regional university with the technicians supplied, we don't have to worry about whether we can get online or not and it's a fantastic facility. And it's good. And it costs us $30, which is so much superior to having to trek to the city every month. (G4-PSY-Female-age 37)*

Balancing geographic isolation against the need for face to face interaction was often accomplished through participation in regional networks. These networks improved access to locally-facilitated CPD, alleviated professional isolation and had a strong effect on both retention and recruitment of allied health professionals to that area.

I knew I wanted to be a sole therapist pretty much from the day I graduated really. I was always interested in rural health. And when this job came up I already knew the OT network in this area is quite phenomenal, really. There's quite a lot of working groups for this CPD side of things, we know each other really well, so you know who to pick up and call in the network if you need to answer questions. They're just a fantastic team to work with. So I'm still here, four and a half years later. (G2-OT-Female-age 33)

Despite their apparent success, management support for regional networks was not common due to pressure to deliver clinical services in a context of severe staff shortages.

We don't get help from management for anything to do it. We just, as a network, want to support each other. (G2-SP-Female-age 31)

Many participants reported that rural high school graduates who moved to metropolitan centres for health professional training were lost to ‘push’ influences such as friendships formed in that setting. Where focus groups were conducted in a town with a regional university offering entry level professional training courses, there was a clear perceived impact on both recruiting rural youth to professional qualifications and retaining them in that region.

I went straight to uni here when I finished high school, and I decided to stay around instead of moving away because my boyfriend is here. (G4-SW-Female-age 23)

### The impact of management

Management was raised most often as a ‘push’ factor, increasing intention to leave. This was a highly charged and frequently raised topic, with two aspects of management being noted. Firstly participants expressed frustration with health service managers responsible for resource management, strategic direction and service models.

For me it's generally about management, and not speech pathology management. It's beyond the level of speech pathology management..it's above and beyond that that makes me get frustrated and want to leave. (G2-SP-Female-age 32)

Pronounced frustration with restrictions on recruitment of vacant positions in the public health system was expressed early in the discussion in every focus group.

In remote centres, where the AH workforce was particularly sparse, participants attributed lack of funding for AH services to the widespread use of nurse managers who may have insufficient background to formulate effective AH service models.

I think it's also de-valuing of allied health…I guess the Hospital in the Home program is a good example. Every other area that's got that program employs an OT and a physio. And there's talk of having one out here, and you suggest that allied health is needed, and you're looked at like you've just grown a horn and it's like, “What?” (G5-OT-Female-age 44)

They don't understand. (G5-MGR-Female-age 44)

So I think that's hard for us that nurses and doctors, because of numbers – run a lot of the health system out here, because that's the greater majority of the work force, so they make the decisions. (G5-PT-Female-age 56)

The second aspect of management influence occurred at the level of clinical or departmental managers. Competent clinical managers were highly valued, especially by younger practitioners and new graduates needing support (Table 6). 

**Table 6:** Major theme and subthemes: Impact of management

**Subthemes**	**Examples**
Management skills	*One thing I've probably learned over the last couple of years that makes people leave is bad management… like there's not flexibility… A bit more transparency about access to PD for managers would be good, but often it's the tiny things that just eventually add up, someone snaps and says, “That's it, I'm out of here. (G2-SP-Female-age 32)*
Clinical support for new graduates	*As a relatively new graduate I was really well-supported… until I got here and then I realized that there was nobody here anymore. There was no support – the structure just really wasn’t there to encourage you to stay. (G4-PT-Male-age 57)*
Support for isolated practitioners	*And I know I’ve got friends who are doing that sort of outreach stuff to smaller towns that have those experiences of being working there for a year, and they’re at the point where they’re like, “I’ve almost had enough of this,” because they just haven’t had that support. (G4-SW-Female-age 24)*
Recruitment	*R1: Health service must support reasonable application for recruitment. What we've had here is a situation where they have illogically said no. Not given any reason, not given a time frame…You're not sure why they're saying no, and if they ever will say yes. That's terrible. That affects morale. That’s the death toll. (G5-MGR-Female-age 45)* *R2: Well the ones that are left here say, “What's the point?” If that’s the way they value the professionals, why bother staying around? Why would I even bother? (G5-OT-Female-age 45)*
Ethical dilemmas	*That’s why I left health services, because operating in the health services was professionally compromising me because you had to be responsible to the system, rather than directly responsible to your client, and quite often the demands of the system are disparate to what the needs of the client are. (G4-PT-Male-age 57)*
Work sector	*What prompted me to move from clinical into academia was the frustration with [our service] going from being managed by [a private company] to being managed by [a public health service]… and I went through 3 directors and I couldn’t do it anymore. And it’s only that I’m still rural and still at [this regional university]. Otherwise I would have left this place altogether. (G4-RAD-Female-age44)*

Clinical managers often took on an advocacy role particularly in relation to CPD access, annual leave, flexible hours, and autonomy.

I find that working in the hospital as an allied health manager with budget constraints… they can cost-contain some of the allied health budget to make up for some of the other areas that they're not able to, and then you're in this dilemma between being able to fight for services, or even for staff, being able to recruit when money is short, and being able to offer the staff that you have adequate professional development when you're being told no, but it seems to be a bit unfair in the way it's actually spread out throughout the hospital. (G6-MGR-Female-age 45)

Support for CPD access was represented by informants as a proxy of overall management support.

### Career progression

All interviewees wanted a better career structure, with advancement being reflected in recognition, advanced work roles, and appropriate remuneration (Table 7).

**Table 7:** Major theme and subthemes: career progression

** Subthemes**	**Example**
Lack of career opportunities	*I think that was a difficulty in keeping people. Up until last year we had no senior positions so if you cut someone out here and they had a level of experience, they had to leave to go to a higher level job. There was nothing, no career structure. (G5-OT-Female-age35)*
Advanced work roles	*You come here and you've got these huge broad naught to death experience, bizarre conditions. You know, people with obscure conditions still live in rural centres and still need to access services, so you get this amazing clinical experience. You don't get pigeon-holed, which is – sometimes it's nice to have – to develop a particular area of expertise, but you still can do that while having very broad enriched clinical load. (G2-SP-Female-age32)*
Remuneration	*… in the city, you know, you can specialize in seeing one type of child, or one type of disorder, and get paid much more than a rural clinician who has to be good at seeing it all. And that does become frustrating, when you know that you have good skills, and you work your butt off to keep your skills, and you can never earn more than –(G2-SP-Female-age 31)*
Recognition	*I think it's recognition, I think it's having that extra challenge, I think it's having the extra opportunity to further your skills because you're expected to do more when you have that label of being a senior… to supervise other staff or go to the quality planning or any of those things…or even just getting that clinical expertise (G6-SW-Male-age 30)*

Motivators for retention varied according to participants’ level of experience. For example, some younger participants highly valued remuneration, and rural practice enabled them to progress upwards in the NSW Health pay structure earlier than would have occurred in metropolitan jobs.

I'm ambitious, you know, I don't want to be a level one forever. I would still be a level one if I had stayed in the city. So it's about, “OK, now I'm here, where's the next step up?” And the career opportunities are all out here, they're not back in the city. (G6-SW-Male-age 29)

Discussion about career progression was frequently expressed in the context of aspirations for advanced work roles and obtaining the training and credentialing required for this type of work.

Well professional recognition, I think, is if you can move into a higher grade position because you've done training, you've done your years of experience… That validates you as a more experienced practitioner. And I mean, of course it's better to be paid a bit more and you feel like you're moving. I think you would feel stagnation if you did the professional development and you didn't get the recognition by moving into a higher wage. (G5-PT-Female-age 56)

Older participants were less concerned with career progression, instead being motivated by type of work and altruism. Many older participants felt that the lack of funded senior positions made it difficult to retain experienced staff in rural areas.

## Discussion

Consistent with the literature, [6,10] personal factors such as life stage, attraction to rural lifestyle, rural origin, integration into the local community [27], employment opportunities for spouses, and a good environment for raising children were important factors in attracting and retaining these participants. The results of this study also suggest that, like nurses, AH professionals are attracted to the social aspects of rural practice [28].

The topic of rural workforce supply is complex and most aspects identified in the data are inter-related. For example, while personal factors are not modifiable, communities can nevertheless play a role in attracting health workers by promoting their region, developing economic and educational infrastructure and fostering culture that supports these values [29]. Locating universities in regional centres to offer qualifying courses can also be effective [30,31].

While the 2010 WHO report on retention of the rural medical workforce makes a conditional recommendation to improve access to CPD [1], this study shows the primary importance of CPD access for AH professionals and is consistent with the limited research literature exploring this topic specifically in the AH professions [32,33].

Obstacles to CPD access include travel costs and time away from work, with the concomitant increase in workloads on return as well as lack of management support [34]. The Australian Nursing and Allied Health Scholarship Support Scheme (NAHSSS), [35] which provides travel subsidy and locum backfill, is an example of evidence based policy encouraging CPD access.

In this cohort, developing regional professional networks was a common, low cost strategy to improve CPD access and reduce professional isolation. While formal face to face CPD courses can also ameliorate professional isolation, [36] the use of distance education through information technology (IT) has equivocal support [37,38]. Use of online or blended education models may require investment in IT infrastructure as well as training in its use [39,40] and distance education pedagogies that address isolation should be investigated.

This study suggests that type of work, CPD access, recognition and remuneration all contribute to career progression and that altruism motivates mature practitioners. Consistent with the literature, [34,41] participants described a flat career structure and a lack of appropriate remuneration as a ‘push’ factor. Nevertheless, our data confirm the relatively low priority attached to financial remuneration in comparison with CPD access and improved communication with managers [42]. Further research on career progression for rural AH professionals may illuminate further strategies to retain experienced staff in the rural context.

Management practices and organisational culture are also modifiable influences that can reduce workforce shortages. Consistent with earlier RAHW survey results, participants perceived there to be an inequitable or ineffective distribution of resources [20]. For example, restrictions on filling vacant positions resulted in high workloads, forcing many respondents to make difficult ethical decisions where equitable access to evidence based treatment was in conflict with fiscal policy [43].

The findings suggest that responsibility for resource allocation divided health service managers from clinicians in an adversarial ‘us’ and ‘them’ relationship. Between these two polarities, clinical managers played a critical role in advocating for appropriate clinical service models and for equitable distribution of available resources. Retention of capable managers is invaluable as they have an essential role in supporting career development and helping clinicians to manage the stressors of rural practice in a climate of scarce resources and high expectations.

Engagement in some clinical practice was important to the managers participating in this study and this should be considered when developing job descriptions for AH management positions. Established senior health service AH advisory positions may improve the equity of resource deployment as well as the usefulness of service models, and could provide a much needed career ladder [44]. Health service management positions should also be open to AH professionals as well as nurses. Allied health professionals who are promoted to management roles may need training in how to manage and develop staff and help supervisees cope with the competing priorities of clinical practice in a context of limited resources [45,46].

A summary of recommendations arising from this research is listed in Table 8.

**Table 8:** Recommendations

***RECOMMENDATIONS***
– *Engage local communities to help attract and retain rural AH professionals*
– *Provide access to universities in regional centres*
– *Increase access to CPD through travel subsidy, locum backfill and management support*
– *Encourage development of regional professional networks*
– *Invest in infrastructure and IT support for online education, and develop blended learning models to deliver CPD while also addressing professional isolation*
– *Support extended practice roles for rural AH professionals, as well as enhancing opportunities for career progression appropriate to life stage*
– *Address workplace culture and provide stress management training & personal support to rural AH professionals*
– *Train allied health managers, and increase their role in decision making for resource deployment and service delivery design*
– *Preserve some access to clinical practice for allied health managers*

It is possible that these results may also have been skewed by a recruitment bias and results may have been affected by the interactive nature of the focus group research method [47]. The choice to maximise focus group heterogeneity in this study, while attempting to identify a common experience, may have resulted in poorer definition of the differences between groups. The generalizability of qualitative research is also problematic and these results need to be assessed in a broader context of the existing literature.

## Conclusions

A number of recommendations to improve recruitment and retention of the rural health workforce are based primarily on research within the medical profession. However, research specifically targeting the AH professions is rare and health policy based on the assumption of transferability between professions may be misguided. Management practices, personal relationships, CPD access and resource allocation were identified themes that are not reflected in the extensive literature on retention of rural doctors.

While this study helps fill the gap in knowledge about recruitment and retention of the rural allied health workforce, further research is needed to distinguish differences between aggregate and single AH data as well as comparing professions that share similar business models, and to assess international transferability of these research results.

## Competing interests

The authors declare they have no competing interests.

## Authors' contributions

SK prepared focus group question guides, conducted the focus groups, analysed the resulting transcripts and coded data. ML and TS validated coding structure through concurrent coding of two manuscripts. SK conducted the literature review and wrote a first draft of the manuscript. All three authors revised and approved the last version of the manuscript.

## Pre-publication history

The pre-publication history for this paper can be accessed here:

http://www.biomedcentral.com/1472-6963/12/175/prepub
